# The Field of Science

**Published:** 1894-06-30

**Authors:** 


### The Field of Science.

Professor Schön has engaged himself on a matter which it is to be hoped will prove of the most significant value to medical science. His field of investigation has been in attempting to isolate from the cultures of pathological micro-organisms, and from the organs of dying patients, such poisons as are characteristic of each affection. Such researches as the scientist has published appear to prove that each micro-organism secretes a particular poison. Patients and animals dying from specific diseases appear to contain certain crystals differing according to the microbe which is the subject of investigation.

"Phthisin" crystals, for example, are so named by Professor Schön as having been found in cases of phthisis. At the same time, although much interest is attached to this particular field of operations, it must be borne in mind that these same crystals have been discovered by other observers, but have been given other names and described under another heading. They were described previously as "altered blood pigments," and even as a contemporary points out, they have been regarded in some cases as charcoal particles which have been absorbed from the lungs.

A timely warning is contained in the pages of a current contemporary with regard to milk, and it is one which it would be well if we bore it more practically in our mind. Too much reliance, we are reminded, must not be placed on the boiling and Pasteurusation of milk. The treatment is absolutely insufficient to rid it of bacterial germs. That is, milk, in spite of such precautionary measures, is not fit to be kept for any considerable time prior to its use. Spores of bacilli may yet be present, in spite of the boiling, which after a short time are capable of developing to such an extent as to cause acidity to the milk, which is then unfit for consumption. Our authority on this subject wisely suggests that persons who are entrusted with the feeding of infants on cow's milk, should be supplied with Litmus Paper, which is a sure and certain test of the acidity of such milk.

The first medical college worthy the name established by the Chinese Government was recently opened with formal ceremony at Tientsin. The project owes its origin to the intelligence and energy of the Viceroy of China and his wife, who constructed the necessary buildings, and placed the direction in the hands of a distinguished graduate of University College, Dublin. Some two well-educated English-speaking students have enrolled themselves, and the work of instruction has already been begun.

Some interesting remarks on thunderstorms were made by the President of the French Meteorological Society on the occasion of a recent meeting of that society. M. Renon said that in some parts of France such storms were of a daily occurrence throughout the yearly cycle, in 1892 no less than 328 being registered during six or seven months. The President contends that thunderstorms are more frequent in Europe than in equatorial regions. As an instance in support of his remarks he quoted the case of Sumatra, where storms occur throughout the six months of the S.E. monsoon, but which are never accompanied by thunder. In the hot regions of the globe M. Renon asserts the thunderstorms are of nearly a stationary character, whereas in France they generally traverse a narrow tract from S.W. to N.E. In Peru they are quite exceptional, and are only known to take place once or twice in a century. In January, 1877, one occurred, but previously to that there had been none since 1803.

